# Medical Diplomates and University Degrees

**Published:** 1904-08-06

**Authors:** 


### Medical Diplomates and University Degrees.

The last few weeks have witnessed the establishment of two new Medical Societies, the London Society of Medical Diplomates, and the Association of Medical Diplomates of Scotland. The former consists, we are told, as ordinary members, of gentlemen who hold diplomas or licenses from either, or both, of the two English Royal Colleges, that is to say, the Royal College of Physicians of London and the Royal College of Surgeons of England, and, as honorary members, of any medical gentlemen of distinction who may be elected to the dignity. The latter is limited to those holding the Diplomas of the Royal Colleges of Physicians and Surgeons of Edinburgh and of the Faculty of Physicians and Surgeons of Glasgow, and its laudable aim is to remove from its members disadvantages which are believed to be unjust, a phrase which presumably implies an intention to claim, for these gentlemen, a social and professional position equivalent to that of University graduates. The London Society goes further, for its avowed object is to obtain for its ordinary members, who, *ex hypothesi*, have neither studied nor graduated at any University, the degree and title of Doctor, to be conferred upon them by some "British University" after a "final examination in medicine"—that is to say, we presume, after an examination from which all other subjects are to be excluded. In other words, the gentleman who possesses only the license of the English Conjoint Board, asks to be enabled to go for three months to a crammer, to present himself for a "final examination in medicine," and to emerge from it so ticketed as to be undistinguishable from one who has graduated in Arts at Oxford or Cambridge as a preliminary to taking his medical degree. We cannot regard this extraordinary proposal with anything but disfavour.

We shall be told, without doubt, that the object of the London Society is to "elevate the medical profession," but we are firmly convinced that no profession and no person ever was or will be permanently "elevated" by a sham. The degree of "M.D." has in England a very special significance. The University which calls a man a "Doctor" certifies, in effect, that he has learnt something sufficiently well to be qualified not only to practise but also to teach it; and this certificate would become a farce if it failed to carry evidence of a sufficient intellectual training as a preliminary to the study of some particular pursuit. It would be fatal to the dignity and status of the medical profession, and to its position in the estimation of the public, if it were to go forth, under the sanction of a British University, that medicine alone, among all other pursuits in which degrees are given, is of such a nature as to be within the intellectual reach of comparatively uneducated people, and to render the usual course of a University education superfluous in the case of those who are intending to engage in it. It would be scarcely less fatal if the medical profession were to be regarded as containing no men who have shown, from the very commencement of their independent lives, a capacity and an intention to occupy leading positions among their brethren. In all callings alike it is felt to be a hardship that persons of good capacity are sometimes kept back by want of means or of opportunities; but we cannot regard it as the proper province of a University to equalise the gifts of fortune, or to place the man who has not studied in a particular manner, however worthy or however capable he may be, upon the same footing as the man who has. The reform which is really required, in the interests alike of the medical profession and of the public, is something very different from a reduction in the value of medical degrees. It is, in truth, a steady elevation of medical education, alike in its preliminary and in its final stages, such as to keep pace with the continual advancement of science, with the increasing difficulty and complexity of the problems which it presents, and with its increasing adaptability to the prevention and cure of disease and to the prolongation of life. The great danger of the present day is that the necessity for a constant improvement of education, as the only means by which the profession can be kept in harmony with its environment, should be overlooked or forgotten. There is, of course, great pressure in this direction from the pupils of the inferior schools. There is pressure from the corporations, who are largely dependent for their incomes upon their position in the license market, and who have lately shown themselves impatient of the control which the Medical Council was intended to exercise. There is also pressure from uninstructed public opinion, which is afraid of definite intellectual superiority in the profession, superiority which guardians of the poor and local sanitary authorities would be unable to resist. By these and similar agencies the necessary reforms will, no doubt, be retarded; but, nevertheless, they must come in time. Perhaps the only thing which could altogether arrest them would be the general bestowal of an eminent title upon men who had done nothing in the direction of earning or deserving it.

